# Infant Red Blood Cell Arachidonic to Docosahexaenoic Acid Ratio Inversely Associates with Fat-Free Mass Independent of Breastfeeding Exclusivity

**DOI:** 10.3390/nu14204238

**Published:** 2022-10-11

**Authors:** Bridget E. Young, Gertrude Kyere-Davies, Jacob W. Farriester, Rohan Varshney, Paul S. MacLean, Nancy F. Krebs, Michael C. Rudolph

**Affiliations:** 1 Department of Pediatrics, School of Medicine and Dentistry, University of Rochester, New York, NY 14642, USA; 2 Harold Hamm Diabetes Center, Department of Physiology, Oklahoma University Health Sciences Center, Oklahoma City, OK 73104, USA; 3 Department of Endocrinology, CU Anschutz Medical Campus, Aurora, CO 80045, USA; 4 Department of Pediatrics, Section of Nutrition, CU Anschutz Medical Campus, Aurora, CO 80045, USA

**Keywords:** infant fat-free mass, AA/DHA ratio, infant RBC membrane composition, lipid mass spectrometry, plasmalogens

## Abstract

The prevalence of childhood obesity has increased nearly ten times over the last 40 years, influenced by early life nutrients that have persistent effects on life-long metabolism. During the first six months, infants undergo accelerated adipose accumulation, but little is known regarding infant fatty acid status and its relationship to infant body composition. We tested the hypothesis that a low arachidonic to docosahexaenoic acid ratio (AA/DHA) in infant red blood cells (RBCs), a long-term indicator of fatty acid intake, would associate with more infant fat-free mass (FFM) and/or less adipose accumulation over the first 4 months of life. The fatty acid and composition of breastmilk and infant RBCs, as well as the phospholipid composition of infant RBCs, were quantified using targeted and unbiased lipid mass spectrometry from infants predominantly breastfed or predominantly formula-fed. Regardless of feeding type, FFM accumulation was inversely associated with the infant’s RBC AA/DHA ratio (*p* = 0.029, R^2^ = 0.216). Infants in the lowest AA/DHA ratio tertile had significantly greater FFM when controlling for infant sex, adiposity at 2 weeks, and feeding type (*p* < 0.0001). Infant RBC phospholipid analyses revealed greater peroxisome-derived ether lipids in the low AA/DHA group, primarily within the phosphatidylethanolamines. Our findings support a role for a low AA/DHA ratio in promoting FFM accrual and identify peroxisomal activity as a target of DHA in the growing infant. Both FFM abundance and peroxisomal activity may be important determinants of infant metabolism during development.

## 1. Introduction

During the first six months of life, both fat-free mass (FFM) and fat mass accumulate at an accelerated rate. Although the mechanisms controlling these processes are not well defined, the nutrients consumed early in life contribute to the way FFM and adipose masses are partitioned. Deposition of excess adipose, or suboptimal accumulation of FFM, is increasingly associated with later life obesity and metabolic disease risks [1,2,3,4]. The human milk (HM) fatty acid profile is tightly correlated with maternal long-term dietary fatty acid (FA) intake, as measured by the FA composition of HM and maternal red blood cell membranes [5,6,7]. We previously found that an elevated omega 6 (*n*-6) to omega 3 (*n*-3) long chain polyunsaturated FA ratio (LC-PUFA; the arachidonic to docosahexaenoic plus eicosapentaenoic acids ratio AA/DHA + EPA) in HM was related to accelerated adipose deposition in the infant [7]. In multivariable models, which included several HM bioactive components, this AA/DHA + EPA n6/n3 LC-PUFA ratio remained positively associated with infant fat mass deposition [8].

In murine models, we found lowering the LC-PUFA AA/DHA + EPA ratio in circulation (in neonatal serum) during the first 14 days of life had no effect on FFM but reduced the litter’s body fat % and produced more numerous, smaller-sized adipocytes in the white adipose tissue, a morphology associated with increased insulin sensitivity [9]. Furthermore, the early life reduction of the LC-PUFA AA/DHA + EPA ratio in the neonatal serum associated with epigenetic regulation (DNA methylation) in white adipose of the proximal promoter for the master adipogenic regulator peroxisome proliferator activated receptor gamma 2 (PPARγ2) and suppressed adipose lipogenic mRNA expression in 14-day-old neonates [9]. These studies combined suggest that variation in infants’ consumption of LC-PUFA n6/n3 ratios may affect partitioning between adipose accumulation and FFM accumulation, which could have long-lasting metabolic programming effects that could influence later-life obesity risk.

Given this previous work, we expanded our over-arching hypotheses about the role of LC-PUFA n6/n3 intake on infant body composition to include infants fed both HM and infant formula. We hypothesized that infants with a 4-month exposure to low AA/DHA ratios (regardless of feeding type) would exhibit bolstered FFM accrual and/or tempered fat mass (FM) accumulation. We selected this time period because early infant weight gain patterns during these first few months represent a powerful predictor of later-life obesity risk [10]. Furthermore, this time period is when infants are consuming an exclusive liquid diet (either HM or infant formula) before solid foods are introduced. Here, we quantified total FA composition of the infant RBCs to calculate the arachidonic to docosahexaenoic acid (AA/DHA) ratio in infant red blood cell membranes (RBCs, erythrocytes) as a long-term indicator of infant fatty acid uptake. We also quantified the individual phospholipids contained in the infant RBCs to understand whether specific phospholipid species correlated with infant growth outcomes such as FFM. Infant RBCs were used because they provide an indication of the infant’s long-term FA uptake regardless of feeding modality (breastfed vs. formula-fed). In both the predominantly breastfed and predominantly formula-fed infants we evaluated, we compared how the AA/DHA LC-PUFA ratio is associated with infant body composition partitioning, and how these phospholipid populations differed between the high and low AA/DHA LC-PUFA ratio infant groups.

## 2. Materials and Methods

All aspects of this study were approved by the Colorado Multiple Institutional Review Board (clinical trials.gov: NCT01693406). Women between 20–36 years of age and pre-pregnant BMI < 40.0 kg/m^2^, carrying a singleton fetus and planning to exclusively breastfeed for at least four months, were recruited and consented during pregnancy. Exclusion criteria included any serious medical disease such as renal disease or diabetes, development of gestational diabetes, pregnancy-induced hypertension, or delivery < 37 weeks of gestation. All women delivered their infants at the University of Colorado Hospital at the Anschutz Medical Campus (Aurora, CO). Maternal pre-pregnancy BMI was based on self-reported pre-pregnant weight and measured height. Normal weight (NW) was defined as a pre-pregnant body mass index (BMI) < 25 kg/m^2^; overweight/obese (OW/Ob) was defined as a pre-pregnant BMI ≥ 25 kg/m^2^. Biological samples were collected at between 4–5 months, before any solid foods were introduced to the infant’s diet.

Breastfeeding Exclusivity. Breastfeeding exclusivity score was calculated at each visit as the percentage of feeds in the previous week that were HM. At 4 months, total breastfeeding exposure was calculated as a percentage to reflect exclusivity and duration [11]. For example, a score of 50% could indicate either exclusive breastfeeding for 2 months and then no breastfeeding for months 2–4, or 50% of feeds from breastmilk over the entire 4-month study [11]. Predominantly breastfed was defined as breastfeeding score > 70%. Predominantly formula-fed was defined as a breastfeeding score < 40%. All formula-fed infants consumed standard (non-hypoallergenic) dairy-based formula made with a vegetable oil blend as the fat source. All formulas were supplemented with DHA (ranging from 15–17 mg/100 kcal) and ARA (ranging from 17–25 mg/100 kcal).

Breastmilk Collections and Infant Body Composition. A fasted, morning, mid-feed HM sample was collected at 4 months, as previously described [11]. In short, a feeding session was initiated by the infant. When the feed was halfway complete, the infant was removed from the breast, the breast cleaned with sterile water and gauze, and a sterile manual breast pump was used to express ~10 mL of HM. Milk was immediately placed on ice and transported to the laboratory where it was stored at −80 °C until analysis. Infant body composition (fat mass, and fat-free mass) was measured by air displacement plethysmography (PEAPOD, Cosmed USA, Inc., Concord, CA, USA), and scale weights were taken at both 2-weeks and 4-months postpartum.

Human RBC collection, lipid extraction, and normalization. Fasting (>8 h) maternal blood was collected via venipuncture and non-fasted infant capillary blood was collected via heel prick at 4–5 months. All blood samples were collected into EDTA tubes and spun at 1000 RCF to separate plasma from red blood cells (RBCs) [7]. RBCs were washed three times using 5 mL cold sterile saline, collected by centrifugation, and saline was decanted before 500 μL of RBC pellet was transferred into cryovials and stored at −80 °C. Lipid extraction was performed according to Rose and Oaklander [12]. Briefly, RBCs were thawed on ice, mixed thoroughly by inversion and 30 μL was added to 400 μL of ultrapure water containing stable isotope internal standards. One milliliter of HPLC grade isopropyl alcohol was added, samples were vortexed vigorously, and incubated at room temperature for 10 min. Five hundred microliters of dichloromethane was added, samples were vortexed vigorously and incubated at room temperature for 5 min. An additional 500 μL of dichloromethane was added and samples were vortexed. To complete phase separation, 500 μL of ultrapure water was added, samples were vortexed and incubated at room temperature for 5 min before centrifugation at 300 RCF for 10 min. FA results were normalized per mg of RBC protein measured by standard BCA assay from the initial resuspension in ultrapure water above.

GC/MS Total Fatty Acid Composition. Total esterified and the non-esterified fatty acid composition in HM and in RBCs were quantified relative to a stable isotope internal standard for each acyl chain-length and saturation using lipid mass spectrometry as previously described [7,9]. The total omega-6 (*n*-6) to omega-3 (*n*-3) ratio (*n*-6/*n*-3), the arachidonic to docosahexaenoic plus eicosapentaenoic acids ratio (AA/DHA + EPA) and the AA/DHA ratios were calculated using the quantitative sum of the *n*-6 fatty acids divided by the sum of the *n*-3 fatty acids [7].

LC-MS/MS and MRM Analysis of Infant RBC Phospholipids. RBC total lipids were extracted as above, with the exception of 1 μL of “SPLASH” Lipidomic Analytical Standard was added (Avanti Polar Lipids) per 1 mL of HPLC grade isopropyl alcohol (e.g., 1 μL per sample), and taken to dryness under gaseous nitrogen, as previously described [13]. Dried lipid fractions were reconstituted by adding 25 μL of solvent B (30:40 hexanes/isopropyl alcohol vol/vol) followed by 75 μL of solvent A (30:40:7 hexanes/isopropyl alcohol/5 mM ammonium acetate) reaching the initial column conditions: 75% solvent A and 25% solvent B. The “SPLASH” Lipidomic Analytical Standard was used to determine the retention times for each of the individual phospholipid classes and to optimize the solvent gradient. Samples were injected into an HPLC system connected to a triple quadrupole mass spectrometer (4000 QTRAP; SCIEX, Framingham, MA, USA) and normal phase chromatography was performed using a silica HPLC column (Ascentis 150 2.1 mm, 5 μm; Supelco, Bellefonte, PA, USA) at a flow rate of 200 μL/min. Solvent B was maintained at 25% for 5 min, increased gradually to 60% in 10 min and then to 95% in 5 min, and was held for 20 min before re-equilibration for 15 min. Mass spectrometric analysis was performed for approximately 600 molecular species of phosphatidic acid (PA), phosphatidylcholine (PC), phosphatidylethanolamine (PE), phosphatidylserine (PS), phosphatidylglycine (PG), phosphatidylinositol (PI), and bis(monoacylglycero)phosphate (BMP) that contained a broad range of fatty acyl groups in the negative ion mode using multiple reaction monitoring (MRM). The ratios between the integrated area of each analyte and the integrated area of the corresponding internal standard for each class were generated in MultiQuant Software (Sciex.com) and normalized to total protein concentration of the RBCs present in each extract as determined by BCA assay. The normalized abundance ratios were subjected to lipid class analysis using customized scripts derived from the OMU package (ver 1.0.4) in the Rstudio environment (ver 4.0.5).

Statistics and Graphical Presentation. Fatty acid quantitative values, their sums, and their quotients for significant differences were calculated using 1-way ANOVA analysis in JMP Pro (ver 15.1; www.jmp.com). Linear regression was utilized to assess the relationship between infant RBC lipid composition variables and infant growth outcomes. Non-normally distributed variables were log transformed to ensure normality of models’ residuals. For models of phospholipid species predicting infant outcomes, a secondary significance level of *p* < 0.0005 was applied to adjust for multiple comparisons. Otherwise, *p* < 0.05 was considered significant. Multivariable modeling was utilized to assess relationships between infant RBC composition variables and infant growth outcomes controlling for infant sex, infant status at 2 weeks, and breastfeeding status. Infant RBC AA/DHA ratio was categorized into tertiles, and differences between infant growth outcome by tertiles were assessed via ANOVA (JMP Pro, ver 15.1). Graphical presentation of fatty acid lipid mass spectrometry plots was done using Prism (ver 9.3; www.graphpad.com). Heatmaps for the fatty acid composition and the phospholipid composition data were created using the Pheatmap (ver 1.0.12) and ggplot2 (ver 3.3.3) packages in the Rstudio environment (ver 1.3.959; R ver 4.0.5).

## 3. Results

### 3.1. Characteristics of the Breastfed and FF Mother and Infant Dyads

Participant characteristics are presented in Table 1. All infants received some breast milk. This cohort consisted of 17 dyads with infants that were nearly exclusively breastfed from mothers with normal weight (BF/NW) or overweight (BF/OW), and five infants that were predominantly formula-fed (FF) and exclusively FF by 4 months. One infant in the breastfed group had a breastfeeding exposure score of 70%; the rest had scores >95%. One infant in the FF group had a breastfeeding exposure score of 38%; the rest had scores < 18%. By design, the breastfeeding exposure was significantly different among groups (*p* < 0.001) and no significant differences were observed by infant sex or for birthweight.

### 3.2. Infant RBC and Breastmilk AA/DHA Ratios Are Related Only in Breastfeeding Dyads

Analyses of the total fatty acid composition present in the HM and the RBCs (long-term indicator of dietary FA intake) of breastfed (BF) infants sampled at 4-months postpartum were performed. The AA/DHA and AA/DHA + EPA n6/n3 ratios between HM and infant RBC FA composition at 4-months postpartum were tightly positively correlated (*p* = 0.0005, R^2^ = 0.56 and *p* = 0.0007, R^2^ = 0.55, respectively, *n* = 17), while the total n6/n3 ratio—predominantly influenced by essential dietary fatty acids (EFA) linoleic and linolenic acid abundance (18: n6 and 18:3 n3, respectively)—did not correlate. The quantitative amount of docosahexaenoic acid (DHA, 22:6 *n*-3, *p* = 0.0038, R^2^ = 0.44) was the only FA that correlated between the HM and infant RBC total fatty acid compositions (Table 2, see Supplemental Data S1 for lipidomics). When maternal RBC and infant RBC within the BF group were analyzed, linoleic acid was highly correlated (18:2 n6; *p* < 0.0001, R^2^ = 0.65), and a trend for linolenic acid (18:3 n3; *p* = 0.098, R^2^ = 0.17) was observed (Supplemental Table S1). Conversely, when all maternal RBC and all infant RBC total fatty acid compositions were considered together, highly abundant palmitic (16:0), linoleic (18:2 n6), linolenic (18:3 n3), and arachidonic (20:4 n6) acids were correlated (each *p* < 0.015, Supplemental Table S1). As expected, only in the breastfeeding dyads were the AA/DHA and the AA/DHA + EPA ratios correlated between mother and infant RBC compositions (R^2^ > 0.43 and *p* < 0.004; Supplemental Table S1). This observation suggests that the bioactive AA/DHA and AA/DHA + EPA ratios present in the RBCs of breastfeeding infants were related to maternal dietary FA intake that was tightly correlated with HM composition consumed by the infant, but not for the infants provided formula that was supplemented with DHA and AA. Together, these results support a causal chain for the bioactive LC-PUFA ratios (i.e., AA/DHA and AA/DHA + EPA) from maternal dietary intake, transmitted through the HM, resulting in the ratios observed in the infant’s RBC total fatty acid composition.

### 3.3. Docosahexaenoic Acid, but Not Other n-3 PUFA, Is Greater in Formula-Fed Infants

**Figure 1:**
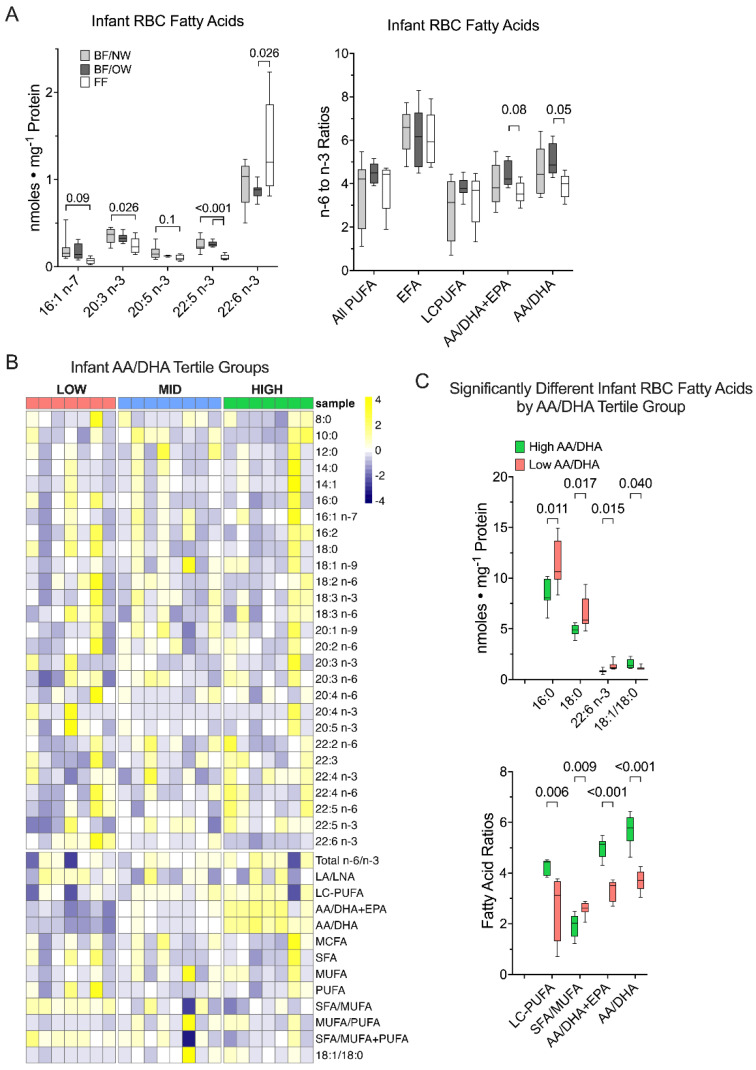
Red blood cell (RBC) fatty acid composition among breastfed and formula-fed infants. (**A**) Infants were grouped by feeding type. In the formula-fed infant group, only docosahexaenoic acid (DHA) was increased, while other key n3 LC-PUFA were decreased or tended to be decreased. Infant RBC total fatty acid levels of arachidonic to docosahexaenoic acid ratio (AA/DHA) were significantly lower in FF relative to the BF/OW infant group (*p* = 0.05 by 1-way ANOVA). (**B**) Heatmap projection of the infant RBC fatty acid composition for each acyl chain length and saturation, indicating a high degree of variance among fatty acids, their quantitative sums, and ratios, when grouped by the RBC total fatty acid AA/DHA ratio. (**C**) Significantly different individual fatty acids or their quantitative ratios when grouped by infant RBC total fatty acid AA/DHA ratio. (BF/NW = breastfed infants/normal weight mothers, BF/OW = breastfed infants/overweight mothers, FF = predominantly formula-fed infants; *n* = 8, 9, and 5, respectively; EPA = 20:5 n3, eicosapentaenoic acid; LC-PUFA = long-chain polyunsaturated fatty acids; SFA/MUFA = saturated to monounsaturated fatty acid ratio; AA/DHA + EPA = 20:4 n6/22:6 + 20:5 n3 arachidonic to docosahexaenoic plus eicosapentaenoic acid ratio; AA/DHA = 20:4 n6/22:6 n3 arachidonic to docosahexaenoic acid ratio).

The infant RBC total fatty acid composition was analyzed because it is a long-term indicator of fatty acid intake among infants that were exclusively breastfed (BF) and those that were predominantly formula-fed (FF) (Figure 1). Overall, the RBC total fatty acid composition from FF infants had only minor significant differences in the quantitative amounts of all RBC total fatty acids measured, relative to the BF group that was further split into maternal pre-pregnancy BMI categories. The quantitative amount of DHA (22:6 *n*-3) was significantly greater in the RBCs of infants in the FF group relative to the BF/OW (*p* = 0.026, Figure 1A). However, the amounts of eicosatrienoic acid (20:3 *n*-3) and docosapentaenoic acid (22:5 *n*-3) were significantly lower in the FF group relative to the BF/NW infants (*p* = 0.026 and 0.001, respectively), while palmitoleic (16:1 *n*-7) and eicosapentaenoic (20:5 *n*-3, EPA) acids tended to be lower in the FF group (*p* < 0.1, Figure 1A). The only significant difference observed in the n6/n3 PUFA ratio was for the AA/DHA ratio (*p* = 0.05) between BF/OW and FF groups, and the AA/DHA + EPA ratio tended to be lower in FF (*p* = 0.08) relative to the BF/OW group (Figure 1A).

### 3.4. Infant RBC AA/DHA Ratio Relationships with Infant Body Composition

Based on our previous findings that the proportions of infant fat-free mass (FFM) and fat mass (FM) accumulation were related to the n6/n3 PUFA ratio present in HM [7,8], we sorted the full cohort of breastfed and formula-fed infant RBCs by the tertile of AA/DHA ratio (Figure 1B). Row-normalized data of the total fatty acid amounts for each acyl chain length and saturation, their ratios, and sums were plotted by heatmap (Figure 1B), demonstrating a high degree of variance among fatty acids when grouping the infant RBC composition by AA/DHA. When grouped by the infant RBC total fatty acid AA/DHA ratio, the saturated palmitic (16:0) and stearic (18:0) acids were significantly increased in the low AA/DHA group (*p* = 0.011 and 0.017, Figure 1C), which led to an increase in the saturated fatty acid to monounsaturated fatty acid proportion (*p* = 0.009, SFA/MUFA), as well as a significant decrease in the oleic acid (18:1 n9) to stearic acid (18:0) ratio. The low AA/DHA group also had significantly less total LC-PUFA (acyl lengths > 20 carbons) in infant RBCs by 4 months of age (*p* = 0.005). DHA (22:6 *n*-3) was significantly increased in the low AA/DHA group (*p* = 0.015), and by design, the AA/DHA (and the AA/DHA + EPA) ratios were highly significantly different (*p* < 0.0001, Figure 1C).

### 3.5. Infant Fat Free Mass Accumulation Is Greater in the Low AA/DHA Ratio Group

**Figure 2:**
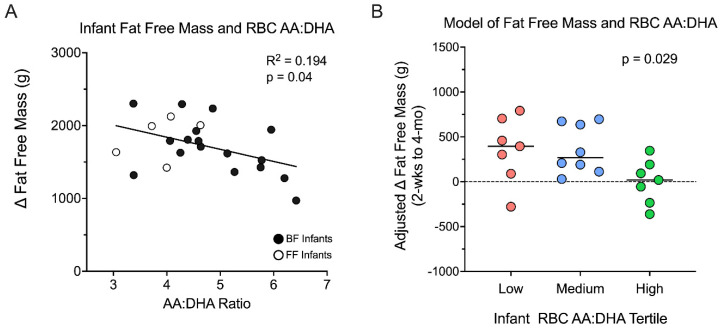
The lower the infant RBC total fatty acid AA/DHA ratio, the greater the infant ∆-FFM/day. (**A**) The change in infant FFM and the RBC AA:DHA ratio is inversely correlated (*p* = 0.04, R^2^ = 0.194), such that as the ratio of AA:DHA in the infant goes up, the less FFM the infant develops. (**B**) Adjusted change in infant FFM in grams is significantly lower in the high AA:DHA infant tertile when controlling for infant sex, infant adiposity at 2 weeks old, and infant feeding type (*p* = 0.029).

Linear regression revealed that infant RBC AA/DHA ratio (as a continuous variable) was inversely associated with the change in FFM (*p* = 0.04, R^2^ = 0.194), indicating that ∆-FFM/day development from 2 weeks to 4 months of age increases as the AA/DHA ratio in the infant RBCs decreased (Figure 2A). When controlling for infant sex, feeding type, and infant adiposity status at 2 weeks old, the ∆-FFM/day between 2 weeks and 4 months remained significantly lower in the high AA/DHA group relative to both the low and middle tertile groups (by 164 g and 95 g, respectively, *p* = 0.029) (Figure 2B). No relationship was observed for indicators of fat mass accumulation, including the change in fat mass per day and the change in % body fat per day, as well as for the overall growth indication of change of weight-for-length z-score per day (Table 3). Taken together, these observations support that the infant’s long-term fatty acid intake containing a lowered AA/DHA ratio may be an important feature in the partitioning of FFM and FM over the first 4 months of human life.

### 3.6. Infant RBC Phospholipid Species Containing AA and DHA Correlate with Changes Infant Fat-Free Mass Accumulation

**Figure 3:**
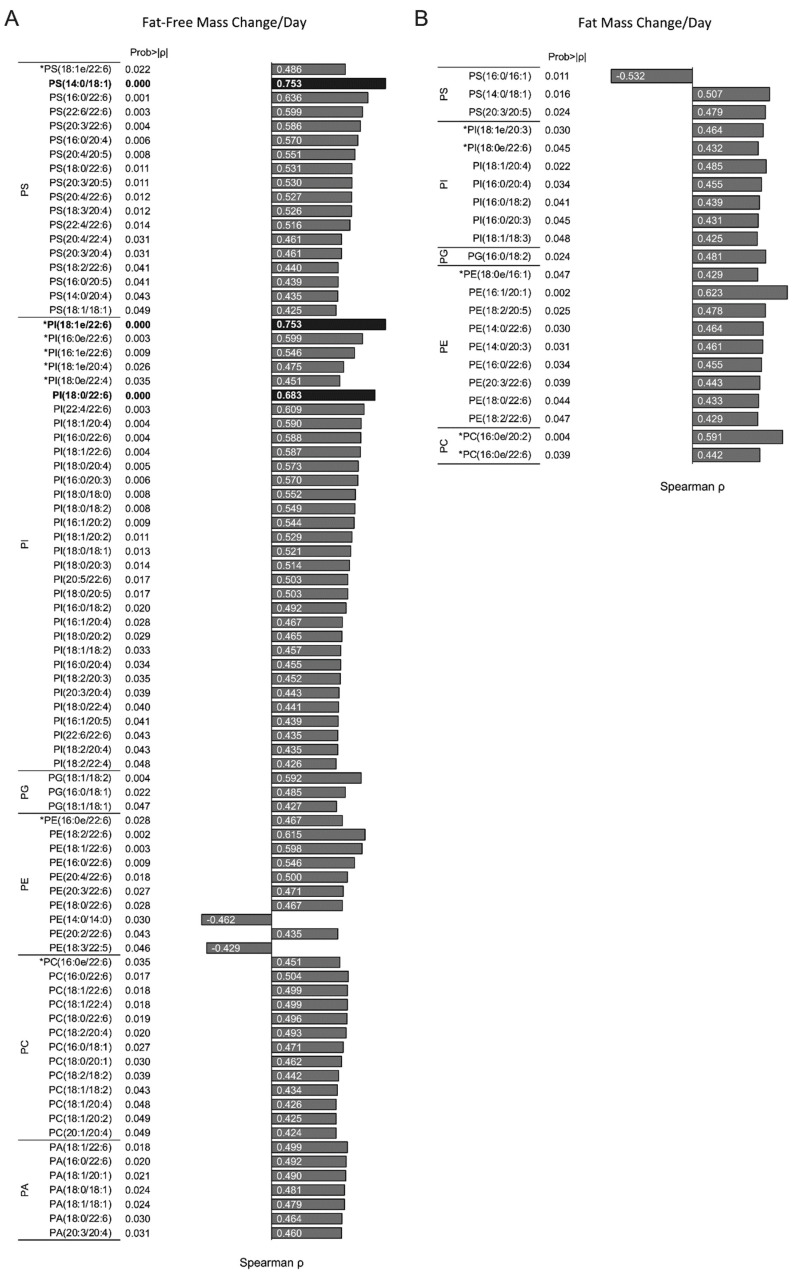
Individual infant RBC phospholipids that correlate with infant ∆-FFM and ∆-FM changes per day. (**A**) Of the nearly 500 individual phospholipids measured, 81 were significantly positively correlated with *p* < 0.05 with ∆-FFM/day, and PS(14:0/18:1), PI(18:1e/22:6), and PI(18:0/22:6) were the most highly significant (boldface yellow). (**B**) Only 22 individual phospholipid species were significantly correlated with infant ∆-FM/day, but none were correlated with *p* < 0.0001 criteria. * denotes plasmalogen phospholipids.

The infant RBC individual phospholipid composition was assessed, and five phospholipid classes including phosphatidylethanolamine (PE), phosphatidylcholine (PC), phosphatidylserine (PS), phosphatidylinositol (PI), and phosphatidic acid (PA) were identified. Within all breastfed and formula-fed infant RBCs, nearly 500 individual phospholipid analytes were reliably detected (Supplemental Data S1). Of them, 83 phospholipids were significantly correlated with ∆-FFM/day (*p* < 0.05; Figure 3A). It is noteworthy how many of these phospholipid species contained either a DHA (22:6) or AA (20:4) or both fatty acid moieties within each phospholipid class (Supplemental Table S2). Three phospholipids remained significantly positively associated with ∆-FFM/day at *p* < 0.0001 (bolded, Figure 3A), two of which contained a 22:6 moiety (PI(18:1e/22:6) and PI(18:0/22:6)). Conversely, 22 phospholipids were significantly correlated with ∆-FM/day (*p* < 0.05; Figure 3B), but none remained significant using *p* < 0.0001 criteria.

### 3.7. Plasmalogens Comprise Half of the Differential Infant RBC Phospholipids by Low AA/DHA Ratio

**Figure 4:**
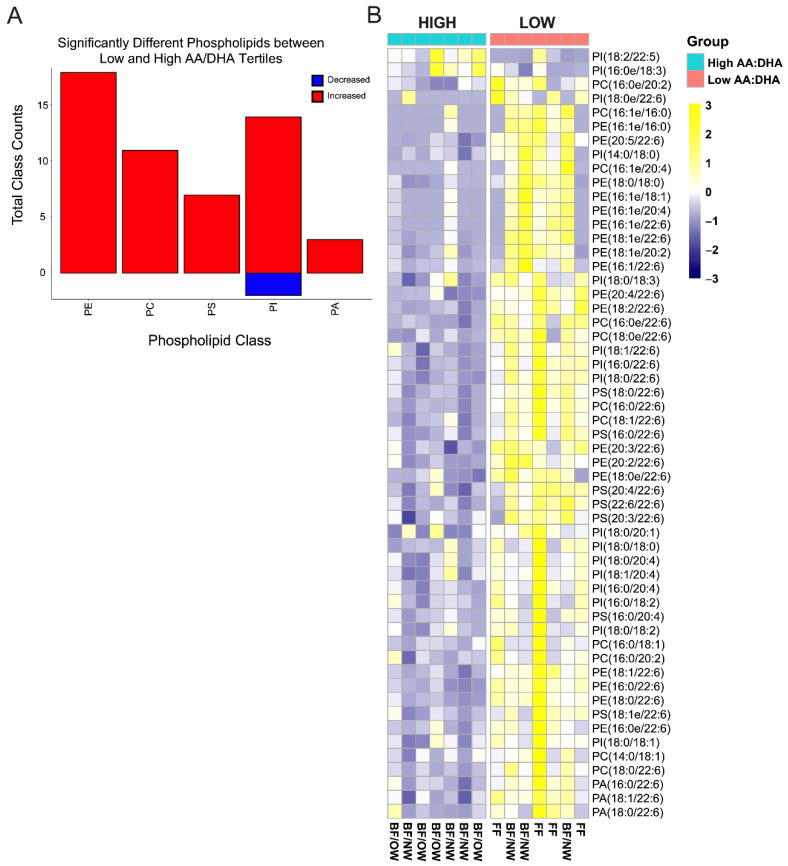
Most significantly different infant RBC phospholipids are increased when grouped by infant RBC total fatty acid AA/DHA ratio. (**A**) Individual classes of phospholipids that are significantly increased or decreased relative to the high AA:DHA ratio and are organized by classes, including phosphatidylethanolamine (PE; 18 increased), phosphatidylcholine (PC; 11 increased), phosphatidylserine (PS; 7 increased), phosphatidylinositol (PI; 13 increased and 2 decreased), and phosphatidic acid (PA; 3 increased). (**B**) Significantly different phospholipids between high and low AA/DHA tertile groups making up each of the phospholipid classes presented in A are plotted by heatmap. (*p* < 0.05 by 1-way ANOVA, Heatmap labels: BF/NW = breastfed infants/normal weight mothers, BF/OW = breastfed infants/overweight mothers, FF = predominantly formula fed infants; *n* = 7 High and 7 Low).

All infants, regardless of feeding modality, were grouped according to the high and low AA/DHA infant RBC total fatty acid ratio to test for differential phospholipid classes between these groups. Five phospholipid classes (PE, PC, PS, PI, and PA) were considered as above. Approximately 11% (56/500) of all individual phospholipids were significantly different between AA/DHA groups. Of these 56 differentially abundant phospholipids, most were increased in the low AA/DHA ratio group relative to the high group (*p* < 0.05, Figure 4A). Relative to the high AA/DHA group, only two phospholipids were significantly decreased: PI(18:2/22:5) and PI(16:0e/18:3). All other phospholipids were increased (Figure 4B). Interestingly, nearly 30% (16/56) of differential phospholipids identified between AA/DHA ratio groups were “ether” phospholipids (plasmalogens, alkyl- glycerophospholipids) (Figure 4B). Of the five FF infants (columns, Figure 4B), four were in the low AA/DHA group, and maternal pre-pregnancy phenotype was reasonably distributed among all AA/DHA groups, suggesting that feeding type might not be entirely responsible for the elevated plasmalogens (ether phospholipids) or the −14 and −16 carbon fatty acids present.

## 4. Discussion

Our findings support the role for the AA/DHA ratio in the patterning of infant body composition during the first 4 months of life. Consistently, infant RBC AA/DHA ratio was related to the ∆-FFM/day between 2 weeks and 4 months. These models suggest that by 4 months, infants that have consumed a low AA/DHA ratio are more likely to develop an increased FFM. Consistent with these results, a double-blind, randomized control trial evaluating *n*-3 PUFA supplementation administered during pregnancy found associations with isometric growth (equal growth of all body components) on childhood outcomes out to 6 years old, suggesting fetal programming by *n*-3 PUFA in humans affects muscle, adipose, and bone mass growth in an equivalent way [14]. However, in adolescents and adults, conflicting evidence exists. Some observational studies link *n*-3 PUFA supplementation with increased FFM accumulation, measured by muscle protein synthesis [15]. Infants with the lowest RBC AA/DHA ratio had greater amounts of saturated FA (16:0 and 18:0, Figure 1C), which are ready substrates for fatty acid oxidation [16] by the mitochondria present in muscle, brown adipose, and beige adipose tissues present in the infant.

Omega-3 PUFA, especially the more bioactive FA containing 22 and 20 carbon chains such as DHA and EPA, are very important contributors to brain [17,18,19] and eye development [20]. Although humans have the capacity to synthesize these bioactive *n*-3 PUFAs from the essential dietary precursor linolenic acid (18:3 n3), the primary source for long chain *n*-3 PUFA is from the diet, especially during infancy [20,21]. All long-chain PUFA (more than 20 carbons; LC-PUFA) are pluripotent, being incorporated into structural lipids (phospholipids), storage lipids (triglycerides), extracellular ligands (GRP120 and GRP40), and modulating nuclear hormone receptor function (PPAR/RXR axis) [20,22,23]. The levels of *n*-3 PUFA and the n6/n3 LC-PUFA ratio early in life have been recognized as a critical component in offspring body composition. For example, diminished amounts of *n*-3 PUFA in the neonate predicted higher risk for elevated adiposity at 3 years [22]. Placental LC-PUFA trafficking during fetal development and the quantitative fatty acid profile of HM have both been related to greater infant adiposity [7,8,24,25]. Importantly, it was recently shown that the DHA level present in infant RBC at 1 month of age, a direct measure of the fatty acid intake by the infant, was inversely related to the infant FM [26].

Peroxisomes are cellular organelles with the primary function to oxidize LC-PUFA and branched chain fatty acids, however, they also play a role in key biosynthetic processes [27]. The current understanding of mammalian DHA synthesis requires FA-modifying enzymes housed in two distinct intracellular organelles. The DHA synthesis pathway begins in the endoplasmic reticulum (involving elongases and fatty acid desaturases), and is completed in the peroxisome (involving acyl-CoA-oxidase for partial beta-oxidation) [28,29]. Conversely, the enzymes that synthesize plasmalogens (ether phospholipids) reside in the peroxisome, and the biosynthetic pathway is completed in the endoplasmic reticulum [30]. Ether phospholipids differ from “conventional” phospholipids (acyl-glycerophospholipids) by the type of covalent bond the ‘fatty moiety’ makes with the glycerol backbone [30]. From a structural perspective, ether phospholipids permit tight packing, resulting in less membrane fluidity, while DHA increases membrane fluidity. Membrane characteristics, including fluidity, are important in processes such as postnatal neural development [20,30,31,32].

The RBC from infants in the low AA/DHA group had a greater portion of plasmalogens (Figure 4, denoted by ‘e’ in the name). This group also had significantly increased 16:0 and 18:0, which can be precursors of ether lipid biosynthesis (Figure 1C), potentially indicating a greater function of the peroxisomal pathway in infants consuming a low AA/DHA food source. It is unclear at present whether the significant differences in plasmalogens arise from biosynthetic differences within the infant, or whether differences are due to exogenous lipid supply (i.e., variances in breastmilk or formula composition). However, when maternal RBC phospholipid data were grouped by the infant’s AA/DHA ratio status, about half of the significantly different phospholipids were in common between the maternal and infant RBC phospholipid differences (Supplemental Figure S1), suggesting a possible genetic linkage in the biosynthetic pathways generating plasmalogens. Altogether, it is interesting to note that a large portion of differential phospholipids between AA/DHA tertile groups (30–40%) were comprised of plasmalogens, especially given the common organelles and biosynthetic machinery necessary for plasmalogen and DHA synthesis.

Increasingly, other lipids present in HM, such as the brown fat-activating oxylipin 12,13-diHOME (signaling lipid), have been associated with decreased infant adiposity [33]. This work confirms that other components within the infant, such as the RBC FA or the RBC phospholipid species composition may associate with infant body composition partitioning and may provide a biomarker for infant metabolism. If infants with a low AA/DHA ratio do indeed have elevated peroxisomal activity, as predicted from the increased plasmalogen levels in their RBC phospholipids, and they may exhibit greater total energy expenditure or a lower respiratory exchange ratio (e.g., fat oxidation). We did not assess infant metabolic rate or oxidative capacity in this study, so this remains an active area of future research.

This study has numerous strengths, including estimation of infant body composition by air displacement plethysmography (PeaPod), inclusion of both breastfed and formula-fed infants, and the combination of HM, infant and maternal RBC lipidomic analyses (including quantitation of total fatty acid composition alongside phospholipid composition). However, this study was limited by a relatively small sample size. This study also identified plasmalogen (ether phospholipids) as increased in the low AA/DHA ratio group, which comprised over 40% of all significantly different phospholipids present in the RBC of the growing infant. Unfortunately, we did not quantify the phospholipid composition of the breastmilk, which could have strengthened the potential causal chain from maternal metabolism to HM to infant blood levels to infant growth outcomes. This study also focused on the first few months of life when exclusive HM/infant formula feeding occurs. Future studies should repeat this line of inquiry in older infants to document how differences in solid food choices may impact RBC fatty acid profile and associated body composition outcomes.

## 5. Conclusions

In this study, we identify a relationship between low AA/DHA ratio in human infant RBC total fatty acid composition and infant accrual of fat-free mass during the initial 4 months of life, regardless of infant feeding type (breastmilk or formula). The low infant RBC AA/DHA ratio was also related to plasmalogen lipids present in the infant RBC phospholipid composition, suggesting a link between peroxisomal function and infant body composition during postnatal development and growth.

## Figures and Tables

**Table 1:** Participant characteristics of mother and infant dyads.

Characteristic	BF/NW (*n* = 8)	BF/OW (*n* = 9)	FF (*n* = 5)	*p*-Value
Maternal age (yrs)	29.9 ± 2.7	31.4 ± 4.4	27.4 ± 4.1	0.1976
Pre-Pregnancy BMI (kg/m^2^)	20.9 ± 2.2	30.5 ± 3.9	33.2 ± 7.1	<0.0001
Gestational age (wks)	39.6 ± 0.8	39.4 ± 0.9	39.6 ± 0.9	0.938
Race	Asian: 12.5% White 87.5%	Asian 1: 22.2% African American: 11.1% White: 66.7%	Hawaiian/Pacific Islander: 20.0% White: 80.0%	0.342
Ethnicity	Hispanic: 12.5% Non-Hispanic: 87.5%	Hispanic: 25.0% Non-Hispanic: 75.0%	Hispanic: 60.0% Non-Hispanic: 40.0%	0.185
Infant sex (% male)	62.5%	77.8%	20%	0.098
Birth weight (g)	3130 ± 357	3473 ± 544	3781 ± 366	0.056
Breastfeeding Exposure (months)	3.9 ± 0.42	4.0 ± 0.06	0.8 ± 0.41	<0.0001

**Table 2:** Correlation values between milk fatty acid composition and Infant RBC at 4-months postpartum among breastfed infants (*n* = 17).

Milk	Infant RBCs	*p*-Value	R^2^
16:0	16:0	0.715	
18:2 n6	18:2 n6	0.397	
18:3 n3	18:3 n3	0.999	
20:4 n6	20:4 n6	0.128	
20:5 n3	20:5 n3	0.997	
22:6 n3	22:6 n3	0.0038	0.44
Total n6/n3	Total n6/n3	0.95	
AA to DHA	AA to DHA	0.0005	0.56
AA to DHA + EPA	AA to DHA + EPA	0.0007	0.55

**Table 3:** One-way ANOVA test for infant lean and fat mass accumulation when grouped by tertiles of infant RBC AA/DHA fatty acid ratio.

Outcome	*p*-Value	Relationship (Tukey)
∆ FFM/day	0.029	high < mid, *p* = 0.032 high < low, *p* = 0.075
∆ Fat Mass/day	0.144	
∆ % Fat/day	0.479	
∆ WLZ/day	0.190	

FFM = fat-free mass; WLZ = weight for length z-score; *n* = 8, 7, and 5/group.

## Data Availability

Data will be made available upon request.

## Supplementary Materials

The following supporting information can be downloaded at: https://www.mdpi.com/article/10.3390/nu14204238/s1. Supplemental Figure S1. Significantly different phosholipids in maternal RBCs are partially in common with differential infant RBC phospholipids. (A) Maternal RBC phospholipids were grouped according to the infant’s RBC total fatty acid AA:DHA ratio (as in Figure 1) and plotted by heatmap, indicating >40% of differential phospholipids within maternal RBCs are also plasmalogens (ether phospholipids). (B) VENN diagram of the overlap between maternal and infant RBC differential phospholipid species. Supplemental Table S1. Correlation analysis between fatty acid composition in Maternal RBC and Infant RBC. Supplemental Table S2. Counts of the phospholipid classes containing 20:4 and 22:6, or both, fatty acyl chains that are associated with infant outcomes. Supplemental Data S1: All GC/MS and LC MS/MS fatty acid and phospholipid composition from infant and maternal RBCs and human milk.

## Abbreviations

Arachidonic acid, AA; docosahexaenoic acid, DHA; eicosapentaenoic acid, EPA; formula-fed, FF; fat-free mass, FFM; fat mass, FM; fatty acid, FA; human milk, HM; long chain polyunsaturated fatty acids, LC-PUFA; red blood cells, RBC.
