# Supplementary material for: Infant Red Blood Cell Arachidonic to Docosahexaenoic Acid Ratio Inversely Associates with Fat-Free Mass Independent of Breastfeeding Exclusivity
Source: Nutrients. 2022 Oct 11;14(20):4238. doi: 10.3390/nu14204238 (PMC9608835; doi:10.3390/nu14204238)
Supplement: Supplementary file 1 [file nutrients-14-04238-s001.zip › Supplemental Tables S1 and S2.pdf]

Supplemental Table S1. Correlation analysis between fatty acid composition in Maternal RBC and Infant RBC.

Maternal FA	Infant FA	All (n=22)		BF (n=17)	
		p-value	R2	p-value	R2
16:0	16:0	<0.0001	0.163	0.386	
18:2 n6	18:2 n6	<0.0001	0.55	<0.0001	0.65
18:3 n3	18:3 n3	0.0036	0.35	0.0979	0.17
20:4 n6	20:4 n6	0.0146	0.263	0.985	
20:5 n3	20:5 n3	0.344		0.345	
22:6 n3	22:6 n3	0.0795	0.146	0.181	
n6 to n3	n6 to n3	0.951		0.649	
AA to DHA	AA to DHA	0.149		0.0038	0.44
AA to DHA+EPA	AA to DHA+EPA	0.184		0.004	0.43

Supplemental Table S2. Counts of the phospholipid classes containing 20:4 and 22:6, or both, fatty acyl chains that are associated with infant outcomes.

Phospholipid Classes	Fat Mass Change	Fat Free Mass Change	Total Correlations
20:4 containing	2	11	13
PA		1	1
PC		1	1
PI	2	4	6
PS		5	5
22:6 containing	6	28	34
PA		3	3
PC	1	4	5
PE	5	7	12
PI		8	8
PS		6	6
20:4 and 22:6 together		2	2
PE		1	1
PS		1	1
Total Lipids	8	41	49
