# Supplementary figures and images for: Infant Red Blood Cell Arachidonic to Docosahexaenoic Acid Ratio Inversely Associates with Fat-Free Mass Independent of Breastfeeding Exclusivity
Source: Nutrients. 2022 Oct 11;14(20):4238. doi: 10.3390/nu14204238 (PMC9608835; doi:10.3390/nu14204238)

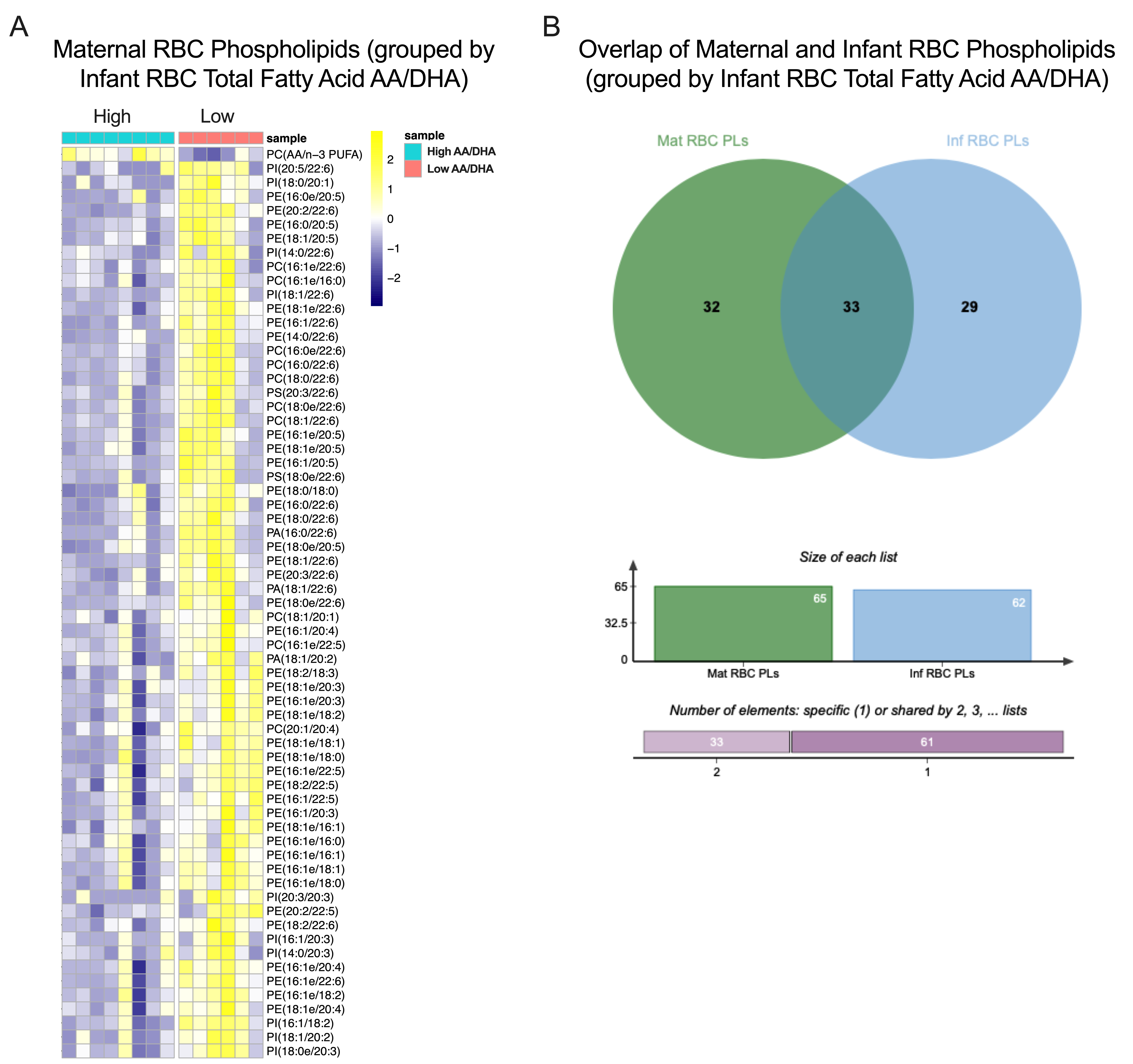

Supplement: Supplementary file 1 [file nutrients-14-04238-s001.zip › Supplemental Figure S1.png]
